# 

**Published:** 1841

**Authors:** 


					THE
AM?IEIE(DAW TOTOWAIL
OP
DENTIL SCIENCE,
FOR
iiTsrcsws'B1 & ssiPEsanEMBs)
1841.
Vol I.?No. XI & XII.
EDITED BY
CHAP IN A. HARRIS, M. D.
Baltimore.
ELEAZAE PARMLY, M. D.
New-York.
PUBLISHING COMMITTEE,
E. PARMLY,M.D.
E. BAKER, M. D.
S. BROWN, M. D.
snsw ?s?<e>3&$8
GEORGE ADLARD, 168 BROADWAY,
ARMSTRONG & BERRY
PRICE?THREE DOLLARS FER ANNUM.
J. A. Fraetas, Pf.inter.

				

